# Anaesthetic Management of a Patient with Multiple Acetyl CoA Dehydrogenase Deficiency: A Case Report

**DOI:** 10.5152/TJAR.2021.816

**Published:** 2022-06-01

**Authors:** Nitu Puthenveetil, Nandhini Joseph, Vijaykumar Dehannathparambil Kottarathil, Jerry Paul

**Affiliations:** 1 Department of Anaesthesiology and Critical Care, Amrita Institute of Medical Sciences, Edappally PO, Ernakulam, India; 2 Department of Gynaeconcology and Breast Cancer, Amrita Institute of Medical Sciences, Edappally PO, Ernakulam, India

**Keywords:** Acetyl coenzyme A, electron-transferring flavoproteins, general anaesthesia, mitochondrial diseases

## Abstract

Multiple acetyl CoA dehydrogenase deficiency is a rare autosomal recessive disorder of amino acid, fatty acid, and choline metabolism. It is a mitochondrial disorder with defective electron transfer flavoproteins or electron transfer flavoprotein dehydrogenases. They are vital for β-oxidation of fatty acids, an essential fuel for skeletal and cardiac muscles. It is also an important source of energy during starvation for the brain. Acute deterioration of these patients can occur during stressful periods like starvation, surgery, infection, and exercise. The anaesthetic management is a challenge with special emphasis on minimizing starvation, ensuring hydration and glucose supplementation, and considering the various effects of anaesthetic agents on the mitochondrial function. The anaesthetic management of a patient with multiple acetyl CoA dehydrogenase scheduled for modified radical mastectomy is described. General anaesthesia can be administered safely in these patients with special emphasis on hydration, glucose supplementation, avoidance of stressors, and monitoring of metabolic status.

**Main Points**

- Multiple acetyl CoA dehydrogenase deficiency is a rare autosomal recessive disorder with significant anaesthetic implications.
- During the administration of anaesthesia, emphasis on hydration, glucose supplementation, avoidance of stressors, and monitoring of metabolic status is paramount.
- Anaesthetic drugs such as induction agents, inhalational agents, neuromuscular blocking agents, and analgesics cause unique challenges.

## Introduction

Multiple acetyl coA dehydrogenase deficiency (MADD) is an extremely rare autosomal recessive mitochondrial disorder of amino acid, fatty acid, and choline metabolism. There is a defect in the electron transfer flavoproteins or electron transfer flavoprotein dehydrogenase enzyme. It has a varied presentation depending on the enzyme activity. This will impair β oxidation leading to the accumulation of medium and long-chain fatty acids and excretion of organic acids in urine.^1,2^ Oxidation of fatty acids is paramount to provide essential fuels to the skeletal and cardiac muscles. This is a vital source of energy for the brain during fasting periods. It is also known as glutaric aciduria (GA) type II.^1,3^ The milder forms do not present with congenital anomalies. They usually present with intermittent episodes of nausea, vomiting, lethargy, weakness and toxicity of liver, central nervous system, and muscle. Acute deterioration can occur due to stress caused by surgery, infection, or exercise. Adequate hydration and prevention of hypoglycemia are important during the perioperative period.^4^ We report the anaesthetic management of a MADD GA type II patient who underwent modified radical mastectomy (MRM).

## Case Presentation

A 42-year-old woman came to the hospital with a lump on the right breast diagnosed as infiltrating ductal carcinoma. She was a known case of MADD GA type II on tablet riboflavin and carnitine since 3 years of age. Her brother also suffered from MADD. Her clinical examination and routine blood investigations were normal. After obtaining written informed consent, she was scheduled as the first case for MRM. She was kept nil by mouth for 2 hours for clear fluids and 6 hours for solids. A maintenance infusion of dextrose containing lactate-free intravenous fluids was started 2 hours before surgery.

The anaesthetic plan was general anaesthesia with laryngeal mask airway (LMA) and spontaneous ventilation. On the morning of the surgery, she was shifted to the operating theatre after ensuring a normal random blood glucose value. Pre-induction monitors included 5 lead electrocardiogram, pulse oximetry, and non-invasive blood pressure. An intravenous (IV) access in the left upper limb was used. She has been induced with IV glycopyrrolate 0.2 mg, IV midazolam 2 mg, IV fentanyl 160 µg, and IV propofol 100 mg. Classic LMA size 3 was inserted. Post-induction monitors included capnography, gas analyzers, and axillary temperature probe. Anaesthesia was maintained using isoflurane, nitrous oxide, and a FiO_2_ of 0.5. Forced air warmers were used to avoid hypothermia. Fluid deficits and requirements were met with IV 0.9% saline. Arterial blood gas (ABG) analysis obtained after induction showed respiratory acidosis with a pH of 7.29 and partial pressure of carbon dioxide of 49.8 mm Hg. The blood glucose was 130 mg dL^−1^, lactate was 1.3 mmol L^−1^, and base excess was −1.34 mmol L^−1^. The surgery was completed in 90 minutes. At the end of the surgery, IV acetaminophen 1 g and IV ondansetron 4 mg were administered. The LMA was removed when the patient was awake and shifted to the recovery room. The ABG and blood glucose tests sent 1 hour after shifting to the post-anaesthesia care unit showed a slight increase in lactate of 2.3 mmol L^−^1. The base excess was −1.68 mmol L^−1^. All other values were within the normal range in the ABG. She was kept nil per oral for 2 hours postsurgically and received IV dextrose-containing fluids during this period. Postoperative analgesia was managed with IV acetaminophen every 8 hours and supplemented with fentanyl boluses. She was sent to the ward after 4 hours and was discharged from the hospital as per protocol.

## Discussion

The perioperative management of patients with MADD causes an anaesthetic challenge with the increased risk of deterioration during surgery. The fasting requirements, stress during the surgery, effects of anaesthetic agents on the mitochondria, fatty acid metabolism, and striated muscles are the main concerns. Currently, there are no guidelines regarding the anaesthetic management of these patients.^4^ Case reports have stressed the importance of ensuring adequate hydration and glucose supplementation.^3-7^

Perioperative fasting transfers energy production to fatty acids through β oxidation. Multiple acetyl coA dehydrogenase deficiency patients are not able to do this effectively resulting in hypoglycemia.^4^ Ideally patients should be posted as the first case to avoid prolonged fasting. We ensured that dextrose-containing fluids were administered during periods of fasting. Since increased lactate load can worsen any metabolic acidosis, lactate-containing fluids should be avoided.^5^

Anaesthetic drugs like propofol, inhalational agents, and neuromuscular blocking drugs pose unique challenges. It is known that most anaesthetic agents impair mitochondrial metabolism in a negative manner.^3,4^ Propofol infusion syndrome (PRIS) characterized by rhabdomyloysis, fatty liver enlargement, metabolic acidosis, and eventual asystole is thought to be due to impaired fatty acid metabolism. However, we used low doses (2-4 mg kg^−1^). At low doses, the concerns of PRIS were negligible.^4-7^ Data suggests that anaesthetic agents like enflurane are known to impair fatty acid metabolism and halogenated agents like sevoflurane impair mitochondrial metabolism in multiple ways. Though there is a theoretical risk in using these drugs, they have not been contraindicated. A risk of malignant hyperthermia has also been raised.^4-7^ Our patient was hemodynamically stable with no elevated temperature.

Muscle relaxants pose a unique challenge in these patients. Some authors recommend avoiding succinylcholine in view of the potential of myopathy. Non-depolarizing muscle relaxants also lead to the risk of hypotonia due to impaired hepatic metabolism.^4,6-7^ We avoided muscle relaxants in our patients and preferred an LMA with spontaneous ventilation.

Opioids except morphine have been described to be safe in these patients.^4^ We used fentanyl and paracetamol for analgesia. Derivatives of propionic acid like ibuprofen, naproxen, and ketoprofen should be avoided.^3^ Acetaminophen is known to cause hepatic toxicity in large doses.^3,5^ It is recommended in doses up to 60 mg kg^−1^ day^−1^ for emergencies.^8^ Case reports have described its safe use.^9,10^

To avoid nausea, vomiting, and shivering, prophylactic antiemetics and forced air warmers were used.

These patients need strict monitoring of glucose, neurological status, and detection of metabolic acidosis. We monitored the ABG and blood glucose levels at regular intervals to assess the metabolic status.

## Conclusion

In patients with MADD, general anaesthesia can be administered with emphasis on adequate hydration, glucose supplementation, and evaluation of metabolic status.
